# Genome Sequence of Bovine Polyomavirus 1 Detected in a Salers Cow (*Bos taurus*) from Catalonia, Spain

**DOI:** 10.1128/genomeA.01658-15

**Published:** 2016-01-28

**Authors:** Nicole Ben Salem, Bernat Pérez de Val, Maite Martin, Ugo Moens, Bernhard Ehlers

**Affiliations:** a Division 12 “Measles, Mumps, Rubella and Viruses Affecting Immunocompromised Patients,” Robert Koch Institute, Berlin, Germany; b Centre de Recerca en Sanitat Animal (CReSA), Institut de Recerca i Tecnologia Agroalimentàries (IRTA), Campus UAB, Bellaterra, Barcelona, Catalonia, Spain; c University of Tromsø, Faculty of Health Sciences, Department of Medical Biology, Tromsø, Norway

## Abstract

We identified a variant of the first bovine polyomavirus (BPyV1; family *Polyomaviridae*) in a lymph node of a Salers cow. As the 2 previously published genome sequences of this virus originated from fetal bovine serum and ground beef, respectively, this is the first BPyV1 genome that could be traced back to an individual.

## GENOME ANNOUNCEMENT

The first bovine polyomavirus (family *Polyomaviridae*) was originally isolated from kidney cell cultures originating from stump-tailed macaque (*Macaca arctoides*). At discovery, it was regarded as a monkey virus and named stump-tailed macaque virus (STMV) (1). Later, independent isolations of polyomaviruses similar to STMV from different monkey kidney cell cultures were reported (2–4). Due to the fact that antibodies against strains of STMV were not detected in monkey but in bovine sera, it was assumed that STMV is of bovine rather than primate origin. Finally, STMVs were identified as contaminants of fetal bovine serum (FBS), a component of the culture medium (5–7). In line with this, STMV was also identified in FBS-free cell cultures from calves (8). In 1990, the genome of STMV (originating from FBS) was sequenced (4697 bp) and the virus was renamed bovine polyomavirus (BPyV or BPyV1; GenBank accession number NC_001442) (9). In a recent study, a variant genome (4746 bp) of BPyV1 (isolate 1S5) was amplified from ground meat collected from a supermarket (accession number KM496323) (10). In that report and in the study of Zhang and colleagues, additional genomes of putatively bovine PyVs (BoPyV2, BoPyV3) were amplified from meat samples (10, 11). However, these are only distantly related to BoPyV1 and not discussed in further detail. Here, we report a complete BPyV1 genome (isolate #7535; 4710 bp) originating from the retropharyngeal lymph node (LN) of a 66-month old female cow (Salers/crossbreed) from a Catalonian cattle farm. The LN was collected in May 2011 after scarification of the animal due to positive diagnosis of bovine tuberculosis. Initially, partial VP1 coding sequence of BPyV1 was identified with generic PCR as described previously (12). Sequences of overlapping PCR fragments (0.9 kb to 1 kb) amplified with primers that had been deduced from conserved regions of the published BPyV1 sequences, were then used to compile a full genome. This was named BPyV1 isolate #7535. It displays 51 and 113 nucleotide (nt) differences to the NC_001442 and KM496323 genomes, respectively. These differences arise from indels in the control region and other noncoding regions and from the absence of 2 codons (Lys^185^, Asp^186^) in the VP1 coding sequence (CDS) of the KM496323 genome. All remaining nt differences represent synonymous base exchanges, with the exception of 2 nt exchanges that cause a Ser^114^ to Asn amino acid exchange in the putative agnoprotein CDS of the #7535 genome and an Asn^104^ to Thr exchange in the large T antigen CDS of the KM496323 genome. The BoPyV1 #7535 genome originates from a naturally circulating virus and is the first that is traceable to a documented cattle individual.

### Nucleotide sequence accession number.

The BPyV1 #7535 genome has been deposited in GenBank under the accession number KU170643.
